# Modeling of Effective Antimicrobials to Reduce *Staphylococcus aureus* Virulence Gene Expression Using a Two-Compartment Hollow Fiber Infection Model

**DOI:** 10.3390/toxins12020069

**Published:** 2020-01-22

**Authors:** Sanjay K. Shukla, Tonia C. Carter, Zhan Ye, Madhulatha Pantrangi, Warren E. Rose

**Affiliations:** 1 Center for Precision Medicine Research, Marshfield Clinic Research Institute, Marshfield, WI 54449, USA; carter.tonia@mcrf.mfldclin.edu (T.C.C.); yzharold@gmail.com (Z.Y.); madhu.pantrangi@preventiongenetics.com (M.P.); 2 Pharmacy Practice Division, School of Pharmacy, University of Wisconsin, Madison, WI 53705, USA; warren.rose@wisc.edu

**Keywords:** mathematical modeling, *Staphylococcus aureus*, antimicrobials, virulence, hollow fiber model

## Abstract

Toxins produced by community-associated methicillin-resistant *Staphylococcus aureus* (CA-MRSA) contribute to virulence. We developed a statistical approach to determine an optimum sequence of antimicrobials to treat CA-MRSA infections based on an antimicrobial’s ability to reduce virulence. In an in vitro pharmacodynamic hollow fiber model, expression of six virulence genes (*lukSF-PV*, *sek*, *seq*, *ssl8*, *ear*, and *lpl10)* in CA-MRSA USA300 was measured by RT-PCR at six time points with or without human-simulated, pharmacokinetic dosing of five antimicrobials (clindamycin, minocycline, vancomycin, linezolid, and trimethoprim/sulfamethoxazole (SXT)). Statistical modeling identified the antimicrobial causing the greatest decrease in virulence gene expression at each time-point. The optimum sequence was SXT at T0 and T4, linezolid at T8, and clindamycin at T24–T72 when *lukSF-PV* was weighted as the most important gene or when all six genes were weighted equally. This changed to SXT at T0–T24, linezolid at T48, and clindamycin at T72 when *lukSF-PV* was weighted as unimportant. The empirical *p*-value for each optimum sequence according to the different weights was 0.001, 0.0009, and 0.0018 with 10,000 permutations, respectively, indicating statistical significance. A statistical method integrating data on change in gene expression upon multiple antimicrobial exposures is a promising tool for identifying a sequence of antimicrobials that is effective in sustaining reduced CA-MRSA virulence.

## 1. Introduction

*Staphylococcus aureus* is a significant human pathogen in both nosocomial and community settings and is capable of causing a variety of infections ranging from skin and soft tissue infections to pneumonia, bacteremia, and osteomyelitis. Many of these diseases are mediated through a variety of virulence factors, particularly toxins. The virulence potential for community-associated methicillin-resistant *S. aureus* (CA-MRSA) primarily comes from a number of known and putative virulence genes [1,2,3]. The virulence profile of *S. aureus* is largely associated with its clonality and, in general, each major clone of *S. aureus* is likely to harbor a similar set of virulence genes [4,5,6,7]. Toxins produced by *S. aureus* can cause outcomes ranging in severity from a high fever to life-threatening toxic shock syndromes and related illnesses [8,9]. Panton-Valentine leukocidin (PVL) remains one of the main toxins present in CA-MRSA and contributes significantly to the pathogenesis of skin and soft tissue infections [10], osteomyelitis [11], and necrotizing pneumonia [12]. Further, expression of PVL in a *S. aureus* USA300 strain in a rabbit disease model resulted in more severe lesions compared to strains lacking PVL [13,14]. Other main virulence factor genes in CA-MRSA are phenol soluble modulins (PSMs), alpha toxin, and, to a smaller extent, toxins made by *sek*, *seq*, *ssl8*, *ear*, and *lpl10* genes [1,4,15,16,17,18,19,20,21,22,23]. PSMs can both have cytolytic activity and be capable of inflammatory response. Alpha toxin, a cytolysin capable of triggering pro-inflammatory response, plays a role in causing pneumonia and skin infections [15]. Staphylococcal enterotoxin K (SEK) is a superantigen and a pyrogen that stimulates CD4+ and CD8+ T cells [24]. SEQ is another staphylococcal enterotoxin with the biological properties of superantigenicity and pyrogenicity [25]. The EAR protein (*Escherichia coli* ampicillin resistance) is a superantigen predicted to have a role in antibiotic resistance due to its partial homology with putative beta-lactamase [21]. In addition, *ear* and *lpl10* genes were observed to be present more frequently in CA-MRSA isolates than carriage or clinical methicillin-sensitive *S. aureus* isolates [4]. The SSl8 toxin is a superantigen that inhibits the tenascin C-fibronectin interaction and cell motility of keratinocytes [26]. Some of the staphylococcal superantigen-like proteins are secreted proteins with roles in immune modulation by binding to immunoglobulins [26].

Many superantigens and superantigen-like proteins can cause tissue damage through an abnormal innate inflammatory cytokine response [8,9]. Patients with CA-MRSA infections are treated with a number of non-beta-lactam antimicrobials but how these antimicrobials affect the expression of some of the virulence genes in CA-MRSA epidemic strains during therapy is not fully understood. We and others have previously shown that antibiotics can reduce and/or regulate the production of virulence factors in vitro, and, in animal models of infection, antibiotics with these properties correlate with improved outcomes [14,16,21,27,28,29]. Antimicrobials are also known to affect *S. aureus* toxin gene expression through transcription and translation [30]. Joo et al. [31] reported that the protein synthesis inhibitor antibiotics, tetracycline and clindamycin, upregulated *agr* and *agr*-controlled phenol soluble modulins.

The aim of this study was to determine the anti-virulence effect of common antimicrobials utilized to treat CA-MRSA USA300-associated infections in a two-compartment hollow fiber model (HFM), mimicking human therapeutic exposures. We modeled the effect of antimicrobials on the expression of well-studied genes such as *lukSF-PV*, which encodes for PVL, and understudied genes such as *sek*, *seq*, *ssl8*, *ear*, and *lpl10* present in the USA300 strain and then applied the same modeling approach to virulence gene expression data for another CA-MRSA strain, MW2.

## 2. Results

### 2.1. Growth Curve of the USA300 Strain in HFM in the Presence of Five Individual Antimicrobials

The USA300 strain was susceptible to all antibiotics evaluated. In the in vitro hollow fiber PK/PD model, clindamycin demonstrated the greatest killing in the first 8 h, but this was not sustained during 24–72 h. Clindamycin growth was equivalent to growth control at the final model time point, demonstrating resistance to clindamycin upon screening (minimum inhibitory concentration > 4 mg/L, Figure S1). This is consistent with inducible clindamycin resistance development reported in some USA300 strains [32]. Similar dose responses occurred with minocycline and SXT, while vancomycin’s effectiveness began to wane after 48 h of treatment and regrew to the initial inoculum. Overall, linezolid sustained the greatest antimicrobial activity over 72 h with up to 99.7% killing (2.5 log_10_ CFU/mL) from the initial inoculum by the end of treatment. Compared to other regimens, linezolid was significantly more active against this organism at 48 and 72 h (*p* < 0.05). The pharmacokinetic targets including validation of predicted and observed concentrations in the hollow fiber model with these regimens have been published previously [16].

### 2.2. Virulence Gene Expression after Antibiotic Exposure

The predicted and calculated pharmacokinetic parameters of the antibiotics from measured concentrations in the hollow fiber model are listed in Table 1. The observed concentrations were within 10% of targeted values of the simulated regimens.

**Figure 1:**
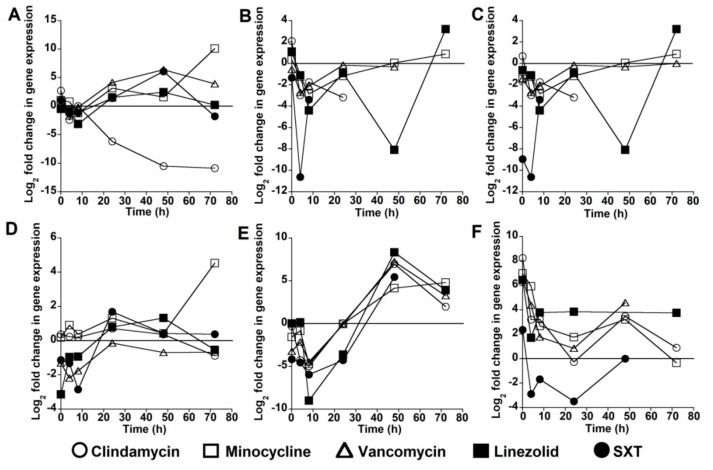
Change in virulence gene expression after antibiotic exposure. Each data point represents the log_2_ fold-change in gene expression after exposure to an antibiotic for the stated time-point compared with expression in the absence of antibiotic exposure for the same time period. Data are shown for: *lukSF-PV* (**A**); *sek* (**B**); *seq* (**C**); *ssl8* (**D**); *ear* (**E**); and *lpl10* (**F**) virulence genes in the USA300 strain.

For each of the six virulence genes in the USA300 strain, expression was reduced after exposure to at least one of the antibiotics tested. No virulence gene showed consistently reduced expression at all time-points after antibiotic exposure. In addition, antibiotics varied in their ability to reduce expression of a given virulence gene. The greatest reduction in *lukSF-PV* expression occurred at T24, T48, and T72 after exposure to clindamycin (Figure 1A). *lukSF-PV* expression at these three time-points was not reduced by any of the other antibiotics except for SXT at T72. The expression of *sek* and *seq* was reduced by several antibiotics at all time-points between T0 and T48, but no reduction in expression was observed at T72 (Figure 1B,C). The expression of *ssl8* was reduced by linezolid, SXT, and vancomycin at T0, T4, and T8, but antibiotic exposure had less of an effect at later time-points (Figure 1D). Antibiotic exposure led to a reduction in *ear* expression at all time-points between T0 and T24, but there was no reduction in expression at T48 and T72 (Figure 1E). The expression of *lpl10* was reduced by SXT at T4, T8, and T24 but not by any of the other antibiotics at any of the time-points (Figure 1F). Gene expression data were not available for some toxins at some time-points: *sek* and *seq* after exposure to clindamycin at T48 and T72, SXT at T24–T72, and vancomycin at T72; *ear* after exposure to SXT at T72; and *lpl10* after exposure to vancomycin at T72, linezolid at T48, and SXT at T72. This was possibly due to gene expression being below the level of detection, as toxin gene expression was able to be detected in control samples unexposed to antibiotics at these time-points.

### 2.3. Optimal Antibiotic Course

**Figure 2:**
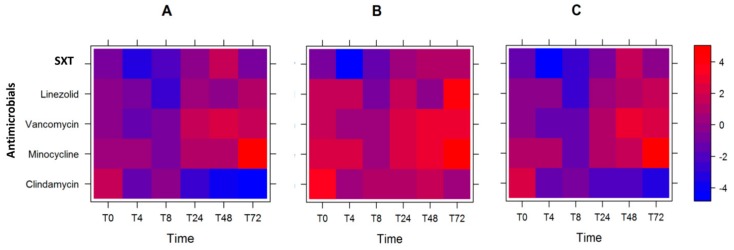
Heat plot showing the optimal course of antibiotics. Heat plot of weighted log_2_ fold-change in expression of the six genes tested, after antibiotic exposure: with the *lukSF-PV* gene given the highest weight (**A**); with the *sel* and *sek* genes given the highest weight (**B**); and with all genes given equal weight (**C**).

Based on the expression data with respect to antibiotic exposure, we sought to model which course of antimicrobials is optimal if we could define the order of preference of virulence gene reduction. We gave a higher weight to *lukSF-PV* (weight = 0.4), followed by the average of *sek* and *seq* (weight = 0.3), *ssl8* (weight = 0.2), and then the average of *ear* and *lpl10* (weight = 0.1). Using this criterion, we observed that the best antibiotics to minimize the log_2_ fold-change of the virulence gene expression levels were SXT at T0 and T4, followed by linezolid at T8, and clindamycin at T24–T72 (Figure 2A and Table 2).

However, when we changed the weight assignments, and *lukSF-PV* was not weighted as the most significant gene (Table 2, weight = 0.0 (for *lukSF-PV*), 0.5, 0.33, 0.17), we observed that the optimum course of antibiotics changed. Here, SXT was the optimum antibiotic from T0 to T24, then linezolid at T48, and clindamycin at T72 for reducing gene expression (Table 2 and Figure 2B). Furthermore, when all the virulence genes were given equal weight (0.25, 0.25, 0.25, 0.25), SXT remained the preferred antibiotic at T0 and T4, followed by linezolid at T8, and clindamycin at T24–T72 (Figure 2C and Table 2). In two of the three scenarios, order of effectiveness of all antibiotics remained the same: SXT, followed by linezolid, and then clindamycin. In addition, we note that the overall log_2_ fold-change of the weighted gene expression levels were increased for all antibiotics, and at most time-points, when *lukSF-PV* was unweighted and *sel* and *sek* genes were given the highest weight (intense red color; Figure 2B) compared to when *lukSF-PV* was weighted highest (Figure 2A) or all genes were weighted equally (Figure 2C). One explanation for this observation is that the reduction of *lukSF-PV* expression is very important to overall reduction of the virulence gene expression levels, which could contribute to the selection of antibiotic use for the USA300 strain.

To determine the statistical significance of the temporal order of the antibiotics described above, we implemented a permutation method to permute the virulence genes expression log_2_ fold-change at given time-points for 100–10,000 simulations and calculated the empirical *p* values, as shown in Table 3. For all permutations for the different weighting approaches, our observations were statistically significant.

### 2.4. Modeling Antibiotic Optimization to Reduce Expression of Selected MW2 Virulence Genes

Despite *S. aureus* strains being largely clonal, it is possible that the same virulence genes in two different clones of *S. aureus* could be regulated differently. Therefore, we also chose to evaluate MW2, the type strain of USA400, a different *S. aureus* clone, to model the effect of antimicrobials on toxin gene expression. We previously reported on the gene expression changes in a MW2 *S. aureus* strain that underwent the same antibiotics simulations in the hollow fiber model [16]. Briefly, five of the genes (*lukSF-PV*, *sek*, *ssl8*, *ear*, and *lpl10*) showed reduced expression after exposure to one or more of the antibiotics, whereas the expression of *seq* was not reduced by any antibiotic after T0. *lukSF-PV* expression was reduced by linezolid, SXT, and minocycline, but, in contrast to the USA300 strain, clindamycin exposure did not lead to reduced *lukSF-PV* expression. The expression of *sek* was reduced by linezolid and SXT at T0 and by vancomycin at T48 and T72, but not by any other antibiotics at other time-points. The expression of *ssl8* and *ear* was reduced after exposure to linezolid and SXT only. The expression of *lpl10* was reduced by linezolid, minocycline, SXT, and vancomycin [16].

**Figure 3:**
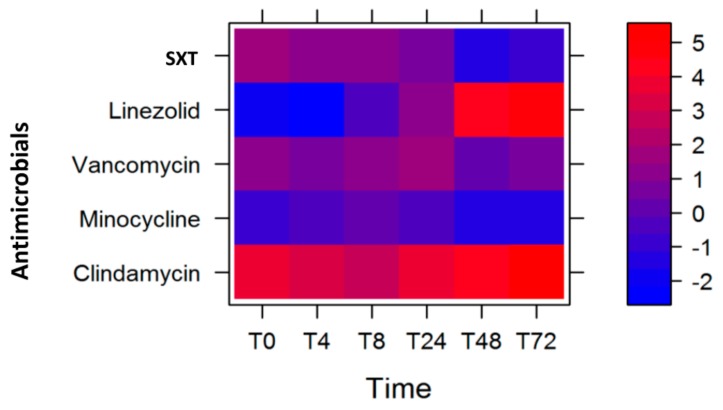
Heat plot showing the optimal course of antibiotics to reduce the expression of the six MW2 genes tested, with the *lukSF-PV* gene given the highest weight.

We performed the statistical modeling and analysis of the MW2 gene expression data with the same antimicrobials, and observed that the largest antibiotic log_2_ fold-change reduction of the select MW2 virulence gene expression levels were with linezolid (T0, T4, and T8) and minocycline (T24, T48, and T72). In this model, we similarly gave a higher weight to *lukSF-PV* (weight = 0.4), followed by the average of *sek* and *seq* (weight = 0.3), next *ssl8* (weight = 0.2), and then the average of *ear* and *lpl10* (weight = 0.1) (Table 4). The corresponding heat plot is displayed in Figure 3.

As in the case of USA300, we sought to determine whether the optimum course of antibiotics for the MW2 strain changed when we changed the weights of the virulence genes. We observed that the course of antibiotics remained unchanged, with linezolid being most effective at T4 and T48 followed by minocycline at T24–T72 (Table 4), despite some changes in the log_2_ fold-change of gene expression levels (represented by the color intensity of red and blue in Figure 3). We further determined the statistical significance of these observations by performing permutations to evaluate the empirical *p* values (Table 5) and noted that our observed course of antibiotics was statistically significant.

## 3. Discussion

Bacterial pathogens often impart damage to the host through the variety of toxins and virulence factors they produce. The goal of antimicrobials in treating bacterial infections is to kill the bacterial cells or inhibit ribosomal transcription and translation of bacterial genes so that they cannot replicate or express the toxin genes. Antibiotics, when given in sub-inhibitory concentrations, may also reduce toxin production through inhibiting protein synthesis or may increase the release of toxins by inhibiting cell wall synthesis [33]. Paradoxically, different antimicrobials can modulate the host immune response differently during inflammation [34,35].

The CA-MRSA USA300 is one such pathogen that produces several virulence factors that are associated with infections of the bloodstream, bones, skin, and tissues. The hollow fiber infection model, a two-compartment in vitro model, could be utilized to mimic the in vivo model to determine the effect of antimicrobials on bacterial growth, virulence expression, and protein production over time.

Our statistical modeling, which was based on giving a weight (or a significance score) to each of the virulence genes, suggested that, over 72 h, a select therapy at particular time-points could be utilized to maximally reduce the expression of priority virulence genes if they are known to contribute to virulence. Based on the quantitative expression of six virulence genes with arbitrarily assigned weights, the sequence of antimicrobials used could be SXT for the first 8 h, followed by linezolid until 24 h, and then clindamycin thereafter. Translating this to clinical doses, this would be one dose of SXT (160 mg trimethoprim/800 mg sulfamethoxazole), followed by one dose of linezolid (600 mg) 12 h later, and then clindamycin (600 mg) every 8 h for the remaining duration. While this was the optimal treatment in our model, future studies could explore the value of simplifying this regimen to SXT followed by clindamycin for improved antibiotic and toxin effects. Interestingly, only the duration of antimicrobial use, but not the sequence of them, changed when the weights of the virulence genes were changed (Table 2). A similar observation was made with respect to modeling with MW2 CA-MRSA, a strain with a different genetic background and virulence arsenal [1,16]. In this case, the sequence of antimicrobials was linezolid until 8 h, followed by minocycline until 72 h, regardless of the weights given to the toxin genes tested (Table 4, Figure 3, and Figure S2A,B). Since recent studies suggest shorter course therapies are important to reduce resistance development, the short duration of each antibiotic in our model to tailor virulence suppression may also have additional benefits.

Based on these experimental data, we suggest that one can use the hollow fiber infection model to assess empirically the effect of antimicrobials on major virulence genes of a pathogen. This approach potentially can help to determine the course of antimicrobials to treat a bacterial infection if the genotype of the suspected bacterial pathogen and some understanding of its virulence are known for major toxin genes. Of future interest is the use of the HFM to understand the anti-virulence effects in *S. aureus* with combination antimicrobial therapy. In addition, these data will be valuable for future mechanistic pharmacodynamic modeling to understand the effects of cumulative drug exposure in the HFM versus a single time-point displayed in this study. Our mathematical model used an agnostic approach to analyze the data and required the experimental conditions to be the same, such as using the same reference gene internal control and data normalization with controls unexposed to the antimicrobials. The study had some limitations. The genes selected for study were limited in scope as not all genes are present in all strains. Future studies will include additional genes including PSMs, alpha hemolysin, and gamma-hemolysin. There was no detectable gene expression of *sek* and *seq* beyond 8 h with SXT exposure and beyond 24 h with clindamycin. The modeling identified the optimal times to use the antibiotics studied; however, it was unable to identify the effects of combination activity in the event of residual drug concentrations from the previous dose when a new antibiotic regimen begins (i.e., residual SXT concentrations from the previous dose when starting linezolid). One of the limitations of the mathematical model in choosing the optimal course was that it was only based on the log_2_ fold-change in expression of the selected virulence genes, and it did not include the role of host immune responses subsequent to a bacterial infection. Although a limitation, the lack of host response allows for the analysis to be focused on the effect of the antimicrobial itself against each bacterial strain of interest. Moreover, the permutation procedure used in this experiment showed a way to evaluate the experimental data objectively and demonstrated the statistical significance of the observed effects of the antibiotic course at all time-points. Future work may include improving the mathematical model to consider the expression of a larger number of genes, quantifying the respective toxin proteins, varying the antibiotic doses, and using longer courses of antibiotic therapy.

## 4. Materials and Methods

### 4.1. Strains

The isolates used in this investigation were CA-MRSA strains USA300-FPR3757 and MW2 (USA400) obtained through the Network on Antimicrobial Resistance in *Staphylococcus aureus* (NARSA) Program supported under NIAID/ IH Contract No. HHSN272200700055C. These strains contain several virulence genes including *lukSF-PV*, *sek*, *seq*, *ssl8*, *ear*, and *lpl10*.

### 4.2. Media

We used Todd Hewitt Broth (THB) (BD Diagnostic System, Franklin Lakes, NJ, USA) growth medium for all susceptibility testing and antibiotic concentration profile simulations in the pharmacodynamic model. This medium has been shown to optimize virulence gene expression and production in vitro [36]. Antibiotic susceptibilities were performed in Mueller Hinton broths supplemented with 25 mg/L calcium and 12.5 mg/L magnesium per Clinical & Laboratory Standards Institute recommendations [37,38], and were compared to susceptibility results in THB to confirm appropriate antibiotic activity in this medium. Bacterial quantification was determined on tryptic soy agar.

### 4.3. Pharmacodynamic Model

We used a two-compartment hollow fiber in vitro pharmacokinetic/pharmacodynamics (PK/PD) infection model to study the effect of antimicrobials on expression of select *S. aureus* virulence genes in a simulated human pharmacokinetics condition. FiberCell hollow fiber C2011 cartridges were obtained from FiberCell Systems Inc. (New Market, MD, USA). This two-compartment capillary system infection model is used to study the effect of simulated pharmacodynamic concentrations of antimicrobials on human bacterial or viral pathogens. This system optimizes drug delivery and allows for simulation of sequestered infections [39]. A single model provides duplicate assessment of antibiotic treatment with sample collection taken from two separate ports of the hollow fiber chamber. Prior to initiating the model, the hollow fiber model was conditioned in THB medium overnight. The inoculum for each model was standardized by injecting 0.2 mL of a 0.5 McFarland bacterial suspension into the 20 mL extra capillary space of the model for a starting inoculum of 1 × 10^6^ colony forming units per mL (CFU/mL). The antimicrobials were administered via bolus infusion after 30 min into the central reservoir of 300 mL to target the free maximum concentration in humans. The unbound free (*f*) antibiotic concentrations were maintained according to the recommended doses and pharmacokinetics for C_max_ and half-life estimates in humans published elsewhere: clindamycin 600 mg every 8 h [40], linezolid 600 mg every 12 h [41], minocycline 100 mg every 12 h [42], SXT—trimethoprim 160 mg/sulfamethoxazole 800 mg—every 12 h [43], and vancomycin 1000 mg every 12 h [44]. The antibiotic regimens were simulated to target pharmacokinetic parameters of C_max_, AUC, and C_min_ and have been previously described [16]. Human pharmacokinetic antibiotic profiles were achieved by adding and eliminating growth medium into the central reservoir to maintain the antibiotic elimination half-life. This model was run in a single replicate with two samples taken for each time point for analysis.

### 4.4. Sample Collection for PK/PD and Gene Expression Studies

Samples were taken before each antibiotic administration from the extra-capillary space of the hollow fiber instrument at 0, 1, 2, 4, 8, 24, 32, 48, 56, and 72 h for PK/PD analysis and at 0, 4, 8, 24, 48, and 72 h for gene expression. Samples for bacterial count determination were serially diluted and quantified for total organisms by spot plating on drug-free agar. The mRNA relative quantification was measured from the same timed intervals during drug exposure, and all samples were drawn in duplicate to account for variability in measurements. The change in colony forming units (CFU/mL) over time was evaluated during antibiotic exposure [45].

### 4.5. Antibiotic Concentration and Half-Life (t_½_) Estimation

In the hollow fiber model, samples were withdrawn from the central reservoir for determination of antibiotic concentrations. The pharmacokinetic parameters were calculated using Phoenix®WinNonLin® pharmacokinetic modeling software (release 5.2; WNL; Pharsight Software, Certara USA Inc., Princeton, NJ, USA). The antibiotic t½ was estimated by linear regression from four or five data points on the initial dose depending on the antibiotic (T0, T1, T2, T4, and T8 for clindamycin plus T12 for all others). Clindamycin, minocycline, and vancomycin concentrations were evaluated by microbioassay using *S. aureus* 6539p as the assay organism as previously described [46]. The interday coefficient of variation of the microbioassays was ±3.6% for each drug. Linezolid and trimethoprim concentrations were determined using high-performance liquid chromatography (HPLC) on a reverse-phase XBridge BEH300 C18 column and XTerra C18 column (Waters Corp., Milford, MA, USA), respectively, as previously described [16]. The limit of quantification was 0.1 µg/mL for linezolid and 0.01 µg/mL for trimethoprim. Sulfamethoxazole concentrations were determined using HPLC with fluorescence detection using an XTerra C18 column (excitation wavelength (λ_exc_) of 267 nm; emission wavelength (λ_em_) of 342 nm) as previously described with a 1.25 µg/mL limit of quantification [16].

### 4.6. Virulence Genes Expression Quantification

We determined the relative quantification of *lukSF-PV*, *sek*, *seq*, *ssl8*, *ear*, and *lpl10* transcripts against an endogenous control gene, gyrase (*gyr*), from the USA300 strain first without antibiotics and then during the simulated exposures. The average gene expression in two replicate samples per model at each time-point was calculated. The mRNA extraction, quantification, and gene expression protocols were described by Pantrangi et al. [17], and the PCR primers and probes were described by Pichereau et al. [16].

### 4.7. Statistical Model and Analysis

The change in gene expression after antibiotic exposure was calculated for a given time-point *t* as Equation (1)

(1) $$A_{tij}=\;log2\;\left(\frac{B_{tij}}{C_{tij}}\right)$$

where $A_{tij}$ is the log_2_ fold-change in gene expression for different antibiotics; $B_{tij}$ and $C_{tij}$ are the average virulence gene expression levels in antibiotic-exposed and control samples, respectively, for the ith virulence gene and jth antibiotic; and *T* is the total number of time-points.

The statistical analyses were designed to identify the antibiotic that produced the largest decrease in virulence gene expression level at each time-point. For a given time-point *t* (Equation (2)),

(2) $$V_{tj}=\overset{N}{\underset{i=1}{\sum }}w_{ij}A_{tij}$$

where $V_{tj}$ is the overall, weighted log_2_ fold-change in gene expression across all virulence genes after exposure to an antibiotic *j*, $w_{ij}$ is the weight and $A_{tij}$ is the log_2_ fold-change in gene expression for the ith gene and jth antibiotic. *N* is the number of virulence genes applied in our model. *M* is the number of antibiotics tested. A single model provides duplicate assessment of antibiotic treatment with sample collection taken from two separate ports of the hollow fiber chamber [1,2,10,16,17,36]. The genes and the order of significance were as follows: *lukSF-PV* (1st), *sek* and *seq* (2nd and considered together), *ssl8* (3rd), and *ear* and *lpl10* (4th and considered together). The weights chosen were 0.4 for *lukSF-PV*, 0.3 for the average of *sek* and *seq*, 0.2 for *ssl8*, and 0.1 for the average of *ear* and *lpl10*. Because the optimal weighting approach was arbitrary, two alternative weighting approaches were also explored: (i) 0.0 (*lukSF*-*PV* was not weighted as being a significant toxin), 0.5 (*sek* and *seq*), 0.33 *(ssl8*), and 0.17 (*ear* and *lpl10*); and (ii) 0.25, 0.25, 0.25, and 0.25, that is, all groups of genes were equally weighted. The use of different sets of weights allowed exploration of how assumptions about significance of the virulence genes affected the model’s results. The aim of the analysis was to find $min_{j\in M}\left\{V_{tj}\right\}$ for each time-point *t*.

A color intensity heat plot was then generated to show for each given antibiotic and time-point the weighted change in gene expression, $V_{tj}$. Assuming that the lower is the weighted log_2_ fold-change in gene expression, the greater is the reduction in virulence gene expression after antibiotic exposure for a given time-point, we plotted the weighted log_2_ fold-change in gene expression in red if the change was positive (increased expression) and in blue if the change was negative (decreased expression). For each time-point, we determined the antibiotic that had the lowest value for the overall, weighted log_2_ fold-change in expression level.

### 4.8. Evaluation of Empirical p-Values

The permutation procedure was based on the experimental data collected and was performed separately for the three weighting approaches used. For each permutation, gene expression was permuted for antibiotic exposures at a time-point *t* to obtain permuted gene expression levels at *t*. This was repeated for each given time-point to obtain the permuted gene expression levels for all time-points. Using the formula (Equation (3))

(3) $$V^{p}{}_{tj}=\overset{N}{\underset{i=1}{\sum }}w_{ij}A_{tij}^{p}$$

we calculated $V^{p}{}_{tj}$, the weighted log_2_ fold-change in gene expression level for antibiotic *j* at time-point *t*. $w_{ij}$ is the weight and $A^{p}{}_{tij}$ is the log_2_ fold-change in gene expression for the ith gene and jth antibiotic. *p* is the number of the permutation. Finally, we determined the best path of $min_{j\in M}\left\{V^{p}{}_{tj}\right\}$ for each time-point *t* and each permutation *p*. This procedure was performed for each permutation *p*.

To evaluate the significance of our observed $min_{j\in M}\left\{V_{tj}\right\}$, we performed *P* numbers of permutations. The permutation method examined whether the optimal sequence of antibiotics based on permuted data was similar to the optimal sequence in observed data by searching for matched time-points between the two datasets, when an antibiotic caused the greatest reduction in toxin gene expression. The empirical *p*-value was calculated as (Equation (4))

(4) $$p-value=\frac{\sum_{i=1}^{P}I\left\{\sum_{t=1\;to\;T}\left[min_{j\in M}\left\{V^{p}{}_{tj}\right\}\;=min_{j\in M}\left\{V_{tj}\right\}\right]=\hat{T}\right\}}{P}$$

Here, T is the total number of time-points and $\hat{T}$ is the number of matched time-points decided by the user. A matched time-point was defined as $min_{j\in M}\left\{V^{p}{}_{tj}\right\}\;=min_{j\in M}\left\{V_{tj}\right\}$ at time-point *t*, and if all time-points between T0 and T72 matched for a permutation *p*, the optimal sequence of antibiotics determined from the permuted data in permutation *p* was the same as that determined from the observed data. The *p*-values were evaluated using two different options for the number of matched time-points, $\hat{T}$. One set of *p*-value evaluations used only the number of times the matched time-points occurred at all time-points between T0 and T72 for a permutation *p*, that is, where $\hat{T}=T$. Another set of *p*-value evaluations were performed allowing mismatch at any one of the time-points between 0 h and 72 h for a permutation *p*, that is, where $\hat{T}=T-1$. The *p*-values were obtained after performing 100, 500, 1000, and 10,000 permutations. All statistics and simulations were done in *R* [47].

## Figures and Tables

**Table 1:** Simulated dosing regimen and targeted and observed pharmacokinetic parameters of the antibiotics in the in vitro hollow fiber model.

Antibiotic	Simulated Dosage Regimen	Half-Life (h)		C_max_ (µg/mL) ^a^	
		Predicted	Observed ^b^	Predicted	Observed ^b^
Clindamycin	600 mg every 8 h	2.4	2.6 ± 0.5	2.8	2.8 ± 0.3
Minocycline	100 mg every 12 h	13.6	13.1 ± 2.1	0.6	0.7 ± 0.1
Linezolid	600 mg every 12 h	7	8.5 ± 0.7	17.1	16.0 ± 0.6
SXT	160/800 mg every 12 h	11/11	10.1 ± 0.3/ 10.9 ± 0.6	0.8/27	0.8 ± 0.1 28.5 ± 1.2
Vancomycin	1000 mg every 12 h	6	6.2 ± 0.7	17	19.2 ± 1.3

^a^ C_max_ = maximum concentration; ^b^ values are mean ± standard error.

**Table 2:** Optimal antibiotic treatment at six time-points with six USA300 virulence genes at each time-point (T).

Weights of Gene	T0	T4	T8	T24	T48	T72
(0.4,0.3,0.2,0.1)	SXT	SXT	Linezolid	Clindamycin	Clindamycin	Clindamycin
(0.0,0.5,0.33,0.17)	SXT	SXT	SXT	SXT	Linezolid	Clindamycin
(0.25,0.25,0.25,0.25)	SXT	SXT	Linezolid	Clindamycin	Clindamycin	Clindamycin

**Table 3:** Number of permutations with USA300 gene expression data.

Number of Permutations	WT = (0.4,0.3,0.2,0.1)		WT2 = (0,0.5,0.33,0.17)		WT3 = (0.25,0.25,0.25,0.25)	
	*P1*	*P2*	*P1*	*P2*	*P1*	*P2*
100	<0.0001	<0.0001	<0.0001	<0.0001	<0.0001	<0.0001
500	<0.0001	0.002	<0.0001	0.002	<0.0001	0.004
1000	<0.0001	<0.0001	<0.0001	0.003	<0.0001	<0.0001
10,000	<0.0001	0.001	<0.0001	0.0018	<0.0001	0.0009

WT means the weights used for expression of each gene. *P1* is the *p* value evaluated using the stringent criteria where no mismatch was allowed for all the time-points of the antibiotic used. *P2* is the *p* value evaluated using relaxed criteria where one mismatch was allowed for all the time-points of the antibiotic used.

**Table 4:** Optimal antibiotic treatment at six time-points with six MW2 virulence genes.

Weights of MW2 Gene	T0	T4	T8	T24	T48	T72
(0.4,0.3,0.2,0.1)	Linezolid	Linezolid	Linezolid	Minocycline	Minocycline	Minocycline
(0.0,0.5,0.33,0.17)	Linezolid	Linezolid	Linezolid	Minocycline	Minocycline	Minocycline
(0.25,0.25, 0.25, 0.25)	Linezolid	Linezolid	Linezolid	Minocycline	Minocycline	Minocycline

**Table 5:** Number of permutations with MW2 gene expression data.

Number of Permutations	WT = (0.4,0.3,0.2,0.1)		WT2 = (0.25,0.25,0.25,0.25)		WT3 = (0,0.5,0.33,0.17)	
	*P1*	*P2*	*P1*	*P2*	*P1*	*P2*
100	0	0	0	0	0	0
500	0	0.002	0	0	0	0.004
1000	0	0.002	0	0.001	0	0.001
10,000	0.0001	0.0014	0	0.0017	0.0001	0.0010

## Supplementary Materials

The following are available online at https://www.mdpi.com/2072-6651/12/2/69/s1, Figure S1: Growth curve of the USA300 strain in the hollow fiber model in the presence of five antimicrobial agents. Figure S2: Heat plot showing the optimal course of antibiotics with MW2 gene expression data with *sel* and *sek* given the highest weight (A) and with all genes given equal weight (B).
