# Supplementary material for: Modeling of Effective Antimicrobials to Reduce Staphylococcus aureus Virulence Gene Expression Using a Two-Compartment Hollow Fiber Infection Model
Source: Toxins (Basel). 2020 Jan 22;12(2):69. doi: 10.3390/toxins12020069 (PMC7076779; doi:10.3390/toxins12020069)
Supplement: Supplementary file 1 [file toxins-12-00069-s001.pdf]

Supplementary Materials: Modelling of Effective Antimicrobials to Reduce *Staphylococcus aureus* Virulence Gene Expression Using a Two Compartment Hollow Fiber Infection Model

Sanjay K. Shukla, Tonia Carter, Zhan Ye, Madhulatha Pantrangi and Warren E. Rose

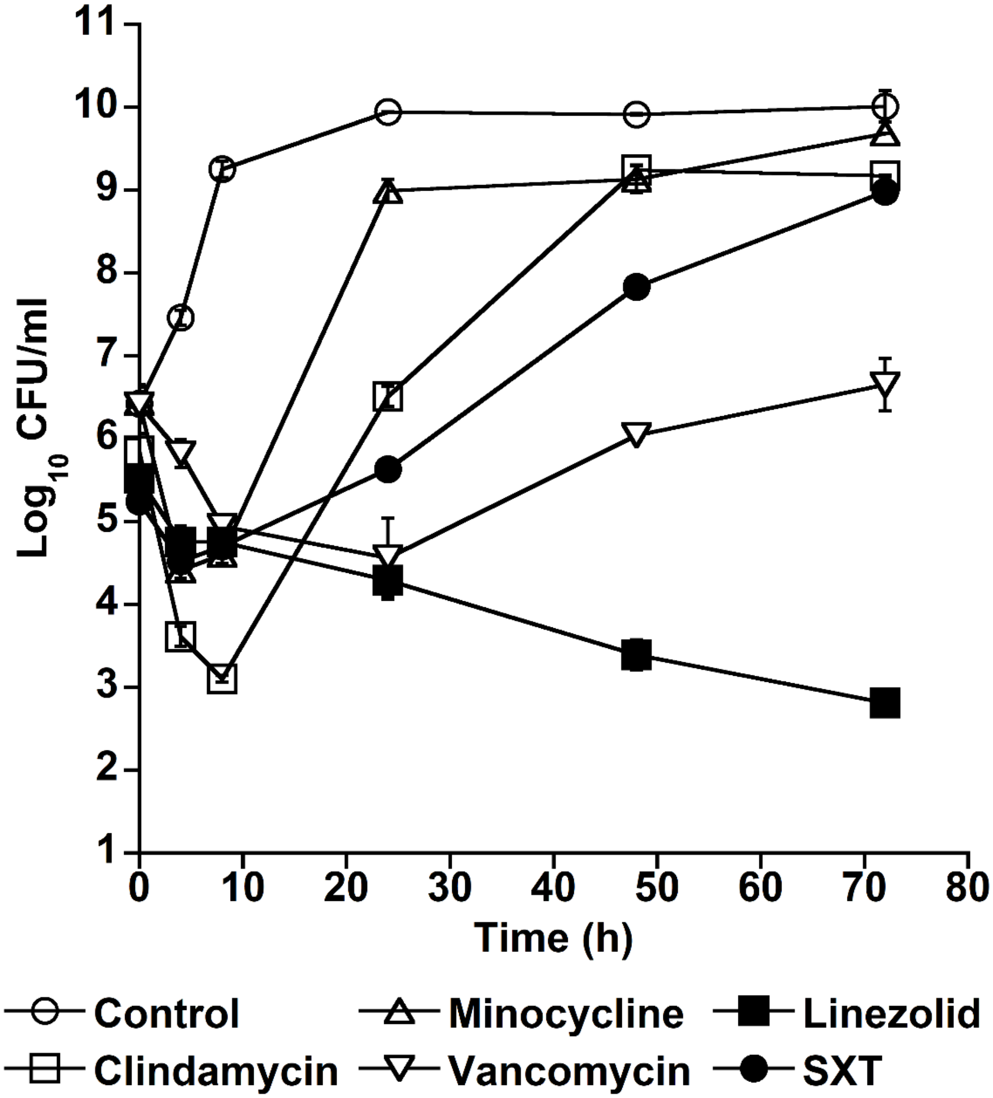

Figure S1. Growth curve of the USA300 strain in the hollow fiber model in the presence of five antimicrobial agents.

(**A**)

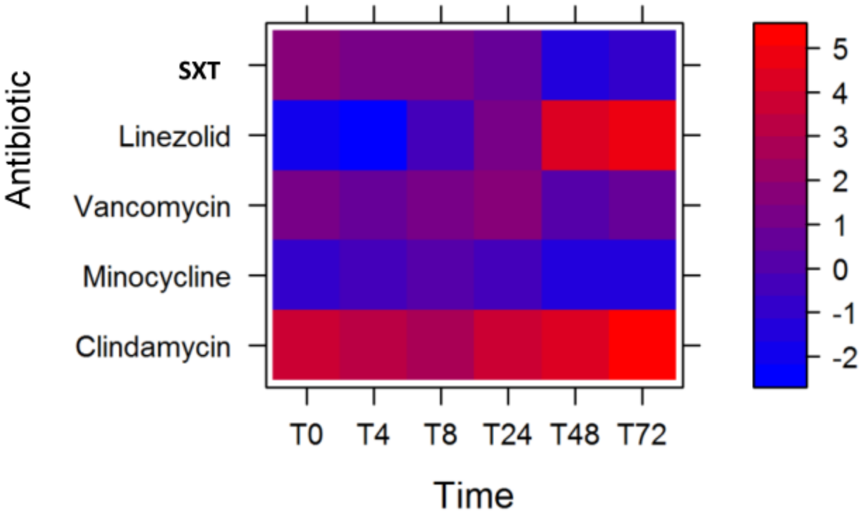

(**B**)

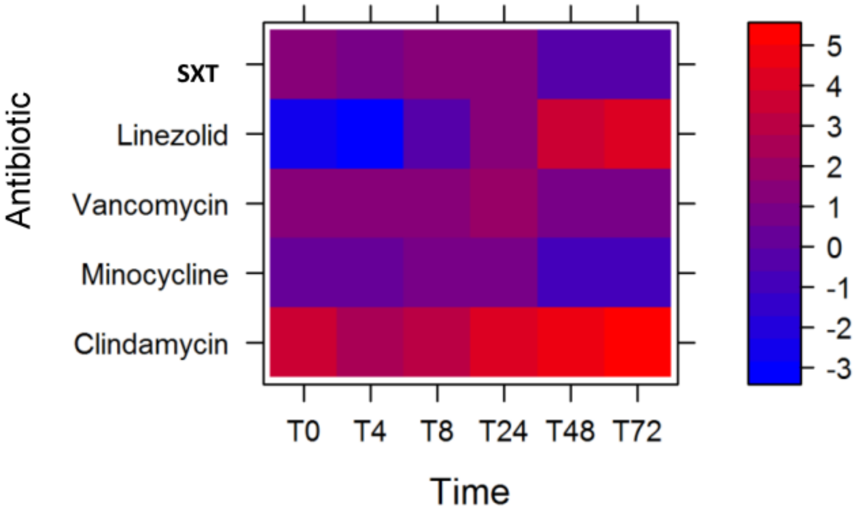

Figure S2. Heat plot showing the optimal course of antibiotics with MW2 gene expression data with *sel* and *sek* given the highest weight (**A**) and with all genes given equal weight (**B**).
